# Redox-Dependent Modulation of T-Type Ca^2+^ Channels in Sensory Neurons Contributes to Acute Anti-Nociceptive Effect of Substance P

**DOI:** 10.1089/ars.2015.6560

**Published:** 2016-08-01

**Authors:** Dongyang Huang, Sha Huang, Haixia Gao, Yani Liu, Jinlong Qi, Pingping Chen, Caixue Wang, Jason L. Scragg, Alexander Vakurov, Chris Peers, Xiaona Du, Hailin Zhang, Nikita Gamper

**Affiliations:** ^1^ Department of Pharmacology, Hebei Medical University, Shijiazhuang, P.R. China.; ^2^ School of Biomedical Sciences, Faculty of Biological Sciences, University of Leeds, Leeds, United Kingdom.; ^3^ Leeds Institute of Cardiovascular and Metabolic Medicine (LICAMM), Faculty of Medicine and Health, University of Leeds, Leeds, United Kingdom.

## Abstract

***Aims:*** Neuropeptide substance P (SP) is produced and released by a subset of peripheral sensory neurons that respond to tissue damage (nociceptors). SP exerts excitatory effects in the central nervous system, but peripheral SP actions are still poorly understood; therefore, here, we aimed at investigating these peripheral mechanisms. ***Results:*** SP acutely inhibited T-type voltage-gated Ca^2+^ channels in nociceptors. The effect was mediated by neurokinin 1 (NK1) receptor-induced stimulation of intracellular release of reactive oxygen species (ROS), as it can be prevented or reversed by the reducing agent dithiothreitol and mimicked by exogenous or endogenous ROS. This redox-mediated T-type Ca^2+^ channel inhibition operated through the modulation of Ca_V_3.2 channel sensitivity to ambient zinc, as it can be prevented or reversed by zinc chelation and mimicked by exogenous zinc. Elimination of the zinc-binding site in Ca_V_3.2 rendered the channel insensitive to SP-mediated inhibition. Importantly, peripherally applied SP significantly reduced bradykinin-induced nociception in rats *in vivo*; knock-down of Ca_V_3.2 significantly reduced this anti-nociceptive effect. This atypical signaling cascade shared the initial steps with the SP-mediated augmentation of M-type K^+^ channels described earlier. ***Innovation:*** Our study established a mechanism underlying the peripheral anti-nociceptive effect of SP whereby this neuropeptide produces ROS-dependent inhibition of pro-algesic T-type Ca^2+^ current and concurrent enhancement of anti-algesic M-type K^+^ current. These findings will lead to a better understanding of mechanisms of endogenous analgesia. ***Conclusion:*** SP modulates T-type channel activity in nociceptors by a redox-dependent tuning of channel sensitivity to zinc; this novel modulatory pathway contributes to the peripheral anti-nociceptive effect of SP. *Antioxid. Redox Signal*. 25, 233–251.

## Introduction

Substance P (SP) is an 11-amino-acid neuropeptide (RPKPQQFFGLM) of a tachykinin family that is produced in both peripheral and central nervous systems (CNS). SP is mainly an excitatory neuromodulator that is involved in pain transmission and smooth muscle contraction, and it modulates inflammatory and immune responses (69). Increased SP release or elevated expression of its receptors has been linked to many diseases such as acute and chronic inflammation and infections, asthma, affective and addictive disorders, and some forms of cancer [reviewed in Steinhoff *et al*. (62)]. In the peripheral somatosensory system, SP is abundantly produced by a subset of transient receptor potential cation channel subfamily V member 1 (TRPV1)-positive, pain-sensing (nociceptive) neurons and released in response to noxious stimulation both centrally and peripherally (40).

Peripheral SP released from nociceptive nerve endings causes so-called “neurogenic inflammation” (54); whereas in the spinal cord, SP functions as an excitatory co-transmitter alongside glutamate (12, 38). Intracellular effects of SP are mediated by the G-protein-coupled neurokinin (NK1 − 3) receptors. The strong excitatory role of SP in spinal nociceptive pathways has triggered a massive, industry-wide quest for new analgesics based on NK1 receptor antagonists. However, despite strong pre-clinical data, clinical trials of NK1 receptor antagonists failed (22, 59).

Innovation We discovered a novel endogenous pathway of “tuning” the activity of T-type Ca^2+^ channels *via* the modulation of its sensitivity to ambient zinc. We further suggest that simultaneous reciprocal modulation of two different ion channels by a single signaling cascade can sum up to produce a cumulative, endogenous anti-nociceptive effect. These findings describe and explain an unanticipated inhibitory effect of neuropeptide substance P (SP) in peripheral nociceptive pathways and shed new light on the mechanisms of endogenous analgesia.

The reasons for the lack of analgesic efficacy of the systemically applied NK antagonists are still unclear. However, one hypothesis is that, though excitatory in CNS, SP may exert paradoxical inhibitory/analgesic effects in the peripheral nociceptive pathways (32, 33). Thus, SP augments an inhibitory M-type K^+^ current in nociceptors *via* the atypical G_i/o_-mediated signaling cascade that utilizes reactive oxygen species (ROS) as second-messenger molecules (32, 33).

Here, we discovered another component of the anti-excitatory action of SP in nociceptors: inhibition of T-type (Ca_V_3) Ca^2+^ channels. Ca_V_3 are encoded by *CACNA1G, CACNA1H*, and *CACNA1I* genes, which give rise to Ca_V_3.1, Ca_V_3.2, and Ca_V_3.3 pore-forming α subunits, respectively (8). T-type channels are activated at voltages near or below −60 mV and may display a significant window current at the neuronal resting membrane potential (E_rest_) (51). Ca_V_3s are abundantly expressed in cell bodies, axons, and peripheral terminals of nociceptive dorsal root ganglia (DRG) neurons, with Ca_V_3.2 being the dominant isoform (9, 11, 18, 56, 63). Accordingly, peripherally applied T-type channel inhibition produces anti-nociceptive effects (2, 65).

Here, we show that (i) SP acutely inhibits T-type Ca^2+^ channels in DRG neurons *via* an ROS-dependent intracellular signaling cascade; (ii) this redox-mediated inhibition operates through the modulation of Ca_V_3.2 channel sensitivity to ambient extracellular zinc; and (iii) a hind-paw injection of SP produces anti-nociceptive effects that can be reduced by the *in vivo* knock-down of Ca_V_3.2 in DRG and partially mimicked by the peripheral injection of a specific T-type channel blocker. Thus, our study establishes a dual mechanism by which SP exerts its peripheral anti-nociceptive effect: a simultaneous inhibition of excitatory T-type Ca^2+^ current and an enhancement of inhibitory M-type K^+^ current.

## Results

### SP inhibits T-type Ca^2+^ current in sensory neurons *via* endogenous ROS generation

SP has been shown to trigger endogenous ROS release in nociceptive DRG neurons in an “atypical” G_i/o_-mediated signaling cascade stimulating mitochondrial ROS generation (33). Since T-type Ca^2+^ channels are expressed in nociceptors and are redox sensitive (49, 65), we investigated whether SP affects the activity of low-voltage-activated (LVA, T-type) Ca^2+^ currents in DRG neurons.

**FIG. 1.**
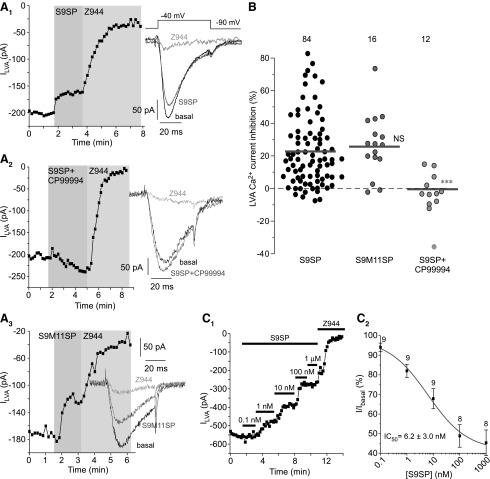
**NK1 receptor activation inhibits T-type calcium channels in small-diameter DRG neurons.** All recordings were performed from small (∼20 μm) DRG neurons with a mean whole-cell capacitance of 23.0 ± 0.9 pF (*n* = 100); these neurons are predominantly (∼70%) capsaicin sensitive (13, 35). **(A)** LVA Ca^2+^ current in DRG neurons is inhibited by an NK1-selective agonist S9SP. **(A_1_)** Example time course of the effects of 1 μ*M* S9SP and 1 μ*M* selective T-type channel inhibitor, Z944 on the LVA Ca^2+^ current recorded from small-diameter DRG neurons using whole-cell patch clamp. Plotted are peak LVA current amplitudes; periods of drug application are indicated by the *vertical gray bars*. *Inset* shows example current traces; current protocol is depicted above the traces. **(A_2_)** Similar to **(A_1_)** but 1 μ*M* S9SP was co-applied with the 3 μ*M* selective NK1 antagonist CP-99994 (after 30 min pre-incubation with the latter). **(A_3_)** Similar to **(A_1,_ A_2_)** but another selective NK1 agonist, S9M11SP (1 μ*M*) was applied instead of S9SP. **(B)** Scatter plot summarizing the experiments exemplified in **(A_1_–A_3_)**. Each *circle* represents LVA current inhibition in a single neuron; the number of individual recordings is given above each group; *horizontal gray bars* are mean values for each group. ***Significantly different from the S9SP group, *p* < 0.001 (unpaired *t*-test). **(C)** Example experiment **(C_1_)** and summary **(C_2_)** of the experiments showing concentration dependency of the LVA current inhibition by S9SP in DRG neurons. Increasing concentrations of S9SP were applied during the periods indicated by *horizontal black bars*. **(C_2_)** The data from eight to nine experiments as in **(C_1_)** were fit to the one-component logistic function (see Materials and Methods section). Data are given as mean ± SEM. CP-99994, (2S,3S)-3-(2-Methoxybenzylamino)-2-phenylpiperidine dihydrochloride; DRG, dorsal root ganglion; LVA, low-voltage activated; NK1, neurokinin 1; S9M11SP, [Sar^9^, Met(O_2_)^11^]-Substance P; S9SP, [Sar^9^]-Substance P; SEM, standard error of the mean; Z944, N-[[1-[2-(tert-butylamino)-2-oxoethyl]piperidin-4-yl]methyl]-3-chloro-5-fluorobenzamide.

Whole-cell recordings were made from cultured, small-diameter, and mostly TRPV1-positive neurons. In 301 of 710 (42%) such DRG neurons, depolarization to −40 mV induced LVA currents, with kinetic properties similar to those of recombinant Ca_V_3.2 (Fig. 1A_1_ *cf.* Supplementary Fig. S1A_1_; Supplementary Data are available online at www.liebertpub.com/ars). These currents were sensitive to the selective T-type Ca^2+^-channel inhibitor, N-[[1-[2-(tert-butylamino)-2-oxoethyl]piperidin-4-yl]methyl]-3-chloro-5-fluorobenzamide (Z944; 1 μ*M*; Fig. 1A_1_–A_3_), and the voltage-gated Ca^2+^ channel (VGCC) inhibitor mibefradil (MIB, 3 μ*M*; Supplementary Fig. S1A_1_–D_1_). In our earlier experiments, we used MIB to confirm current identity but later switched to Z944, when it became available.

The mean LVA current amplitude was −161.7 ± 11.3 pA. The NK1-specific agonist [Sar^9^]-Substance P (S9SP; 1 μ*M*) acutely inhibited LVA current in the majority of the LVA-expressing DRG neurons (Fig. 1A_1_, B); the mean inhibition was 22.5% ± 2.3% (*n* = 84; *p* < 0.001), although there was a degree of scattering in the magnitude of a response and approximately a third of neurons tested displayed minimal response (*e.g.*, 28/84 or 33% neurons had <10% inhibition), which was suggestive of a low level of NK1 receptor expression in these neurons.

Analysis of the concentration dependency of S9SP effects (Fig. 1C) revealed that SP inhibited LVA current in DRG neurons, with IC_50_ of 6.2 ± 3.0 n*M* and maximal inhibition of 59.2% ± 4.2% (*n* = 8–9). NK1-selective antagonist (2S,3S)-3-(2-Methoxybenzylamino)-2-phenylpiperidine dihydrochloride (CP-99994) abolished the effect of S9SP (Fig. 1A_2_, B). Another NK1-selective agonist, [Sar^9^, Met(O_2_)^11^]-Substance P (S9M11SP; 1 μ*M*) produced LVA current inhibition quantitatively similar to that of S9SP (Fig. 1A_3_, B).

**FIG. 2.**
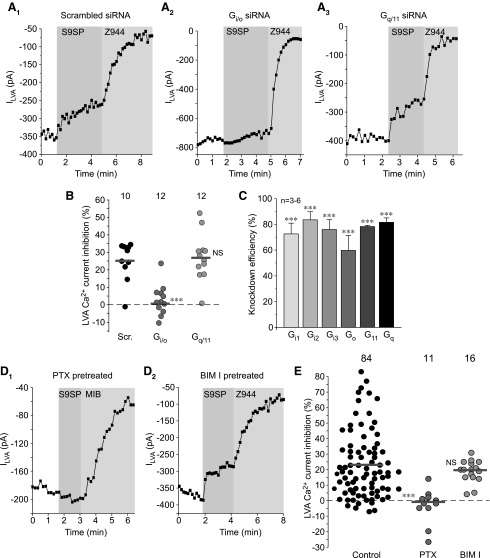
**NK1 receptors inhibit LVA currents in DRG neurons using a G_i/o_-mediated signaling cascade. (A)** Example time courses of the effects of S9SP in DRG neurons where either G_i_ and G_o_ **(A_2_)** or G_q_ and G_11_ **(A_3_)** Gα subunits were knocked down using siRNA cocktails (see Materials and Methods section). Non-targeting “scrambled” siRNA oligonucleotides were used as negative control **(A_1_)**. **(B)** Scatter plot summarizing the experiments exemplified in **(A_1_–A_3_)**. Each *circle* represents LVA current inhibition in a single neuron; the number of individual recordings is given above each group; *horizontal gray bars* are mean values for each group. **(C)** Quantification of knock-down efficiency 48 h post-transfection from experiments as these shown in **(A, B)**. Data are given as mean ± SEM; ***Significantly different from the scrambled oligo control, *p* < 0.001 (unpaired *t*-test). **(D)**. The G_i/o_ inhibitor PTX but not the protein kinase C inhibitor BIM I inhibited NK1-induced LVA Ca^2+^ current inhibition. Example time courses of the effects of 1 μ*M* S9SP and 1 μ*M* Z944 on the LVA currents recorded from small-diameter DRG neurons after the PTX (400 ng/ml; overnight; **D_1_)** or BIM I (200 n*M*; 20 min; **D_2_**) pretreatment. Plotted are peak LVA current amplitudes; periods of drug application are indicated by the *vertical gray bars*; VGCC inhibitor MIB (3 μ*M*) was used instead of Z944 in **(D_1_)**. **(E)** Scatter plot summarizing the experiments exemplified in **(D_1_, D_2_)**, similar to that shown in **(B)**. ***Significantly different from the control, *p* < 0.001 (unpaired *t*-test). The data for the S9SP inhibition in control conditions (*black circles*) are the same as in Figure 1B and are included for comparison. BIM I, bisindolylmaleimide I; MIB, mibefradil; PTX, pertussis toxin; siRNA, small interfering RNA; VGCC, voltage-gated Ca^2+^ channel.

Although NK1 receptor signaling is generally associated with the G_q/11_ type of signaling cascade (37, 43), recent data suggest that at least some effects of SP in DRG neurons, such as ROS release and redox-dependent M channel augmentation, occur *via* the G_i/o_-associated pathway (33). To test which signaling cascade is used in the S9SP-mediated inhibition of LVA current in DRG neurons, we used small interfering RNA (siRNA) knock-down of either G_i/o_ or G_q/11_ Gα subunits in DRG cultures (Fig. 2A). Simultaneous knock-down of G_i1–3_ and G_o_ subunits abolished the S9SP-mediated LVA current inhibition, whereas simultaneous knock-down of G_q_ and G_11_ subunits was without an effect (as compared with non-targeting “scrambled” siRNA control; Fig. 2A, B). All siRNA oligos reduced the abundance of their corresponding target messenger RNA (mRNA) by 60%–80% (Fig. 2C).

In accord with these findings, overnight pre-treatment of DRG cultures with the G_i/o_ inhibitor, pertussis toxin (PTX; 400 ng/ml) completely abolished the S9SP-mediated T-type current inhibition (Fig. 2D_1_, E). A protein kinase C inhibitor, bisindolylmaleimide I (BIM I, 200 n*M*) was without a significant effect (Fig. 2D_2_, E). These data indicate that, similar to their effect on the M channels, NK1 receptors use the G_i/o_-mediated pathway to inhibit T-type channels in DRG neurons. Immunohistochemical localization of Ca_V_3.2, NK1, and a major M channel subunit, KCNQ2 revealed that, indeed, Ca_V_3.2 co-localizes with NK1 and KCNQ2 in many DRG neurons, particularly in those of a small diameter (Supplementary Fig. S2).

**FIG. 3.**
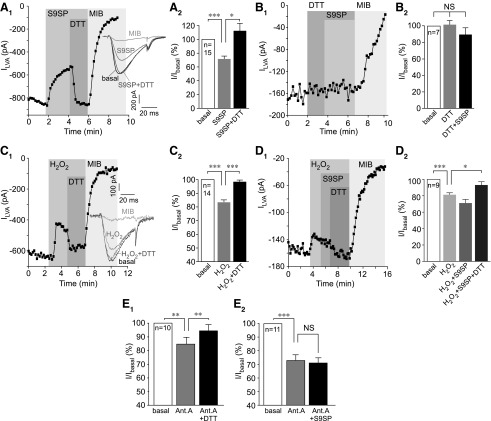
**SP inhibits T-type calcium channels using a redox-dependent mechanism. (A)** S9SP-induced inhibition of LVA current in DRG neurons is reversed by the reducing agent DTT. **(A_1_)** Example time course of the effects of 1 μ*M* S9SP, 1 m*M* DTT, and 3 μ*M* MIB on the LVA Ca^2+^ current recorded from small-diameter DRG neurons. *Inset* shows example current traces; the current protocol was similar to that shown in Figure 1A_1_. **(A_2_)** Summarizes the effects. **(B, B_1_, B_2_)** DTT prevents the SP-induced LVA current inhibition. Experiments are similar to those shown in **(A),** but DTT is applied both before and during the S9SP application; layout and labeling are similar to **(A)**. **(C)** Example time course of the effect of H_2_O_2_ (300 μ*M*) on LVA Ca^2+^ currents in small-diameter DRG neurons. Note that DTT (1 m*M*) could almost completely reverse the inhibition. **(C_2_)** Summarizes the data for experiments shown in **(C_1_)**. **(D_1_, D_2_)** Pre-application of H_2_O_2_ precludes the effect of S9SP on LVA Ca^2+^ currents in small-diameter DRG neurons. Layout and labeling are similar to **(A–C)**. **(E)** Summarized data of the effect of the mitochondrial electron transport chain inhibitor, Ant.A (1 μ*M*) on the LVA currents in DRG neurons. **(E_1_)** Ant.A inhibited LVA current, an effect that was completely reversed by DTT (1 m*M*). **(E_2_)** S9SP applied after the Ant.A produced only marginal further inhibition of LVA current. The number of experiments is shown within the bars. *Asterisks* indicate significant difference from the group indicated by the *line connector* with *p < 0.05, **p < 0.01, or ***p < 0.001 (paired *t*-test). In all bar charts, data are shown as mean ± SEM. Ant.A, antimycin A; DTT, dithiothreitol; SP, substance P.

Washout of S9SP (up to 5 min) did not restore LVA current amplitude (not shown) but the inhibition was completely reversed by the reducing agent dithiothreitol (DTT; 1 m*M*), even in the continued presence of S9SP (Fig. 3A); DTT on its own did not significantly affect LVA current amplitude, but it rendered S9SP ineffective when both compounds were applied together (Fig. 3B).

S9SP had a similar effect on recombinant Ca_V_3.2 (main T-type channel subunit in DRG) that was transiently overexpressed in human embryonic kidney (HEK293) cells together with NK1 receptors (Supplementary Fig. S1A). The inhibition of T-type channels by S9SP was accompanied by a modest leftward shift of the Ca_V_3.2 voltage dependence (Supplementary Fig. S3A_1_) and a small but significant slowing of the inactivation kinetics (Supplementary Fig. S3A_2_). In accord with previous findings (58), in DRG neurons, S9SP had no significant effect on the high-voltage-activated (HVA) Ca^2+^ currents that are mainly conducted by N-, P/Q-, and L-type VGCCs (55) (Supplementary Fig. S3B).

Reversibility of the S9SP-induced T-type current inhibition by DTT suggested a redox-related mechanism. Thus, we investigated whether exogenous or endogenous ROS can produce action similar to S9SP on the channels. Bath application of H_2_O_2_ (300 μ*M*) inhibited LVA current in cultured DRG neurons by ∼20% (Fig. 3C, D; scatter plots for inhibition in each individual neuron in this and other similar experiment series are shown in Supplementary Fig. S4). The effect of H_2_O_2_ was also reversed by DTT. Application of S9SP after (and in the presence of) H_2_O_2_ did not produce a significant further inhibition of the LVA current (Fig. 3D). H_2_O_2_ also inhibited recombinant Ca_V_3.2, an effect that was reversed by DTT (Supplementary Fig. S1B).

The mitochondrial electron transport chain (ETC) is one of the main sources of intracellular ROS (70), and SP was shown to induce ROS release from mitochondria in DRG neurons (33). Inhibition of the ETC complex III with antimycin A has been shown to cause a burst release of mitochondrial ROS (76), and antimycin A-induced ROS release augmented the activity of redox-sensitive M-type K^+^ channels in DRG neurons (33). Consistently, application of antimycin A (1 μ*M*) inhibited LVA current in DRG by ∼25% (Fig. 3E), and this effect was completely reversed by DTT (Fig. 3E_1_). S9SP applied after the pre-application and in the continuous presence of antimycin A only had a marginal further effect (Fig. 3E_2_).

These experiments suggest that a major component of S9SP-mediated T-type channel inhibition is mediated by endogenous ROS (although a contribution from additional mechanisms cannot be excluded).

### SP acts by increasing Ca_V_3.2 sensitivity to ambient zinc

Recently, we showed that hydrogen sulfide (H_2_S) inhibits Ca_V_3.2 by increasing channel sensitivity to zinc (15), a trace metal that inhibits Ca_V_3.2 at sub-micromolar concentrations and is present in sufficient amounts in biological fluids to affect Ca_V_3.2 activity (27, 66). Therefore, we tested whether a similar mechanism might account for the SP-mediated T-type channel inhibition.

**FIG. 4.**
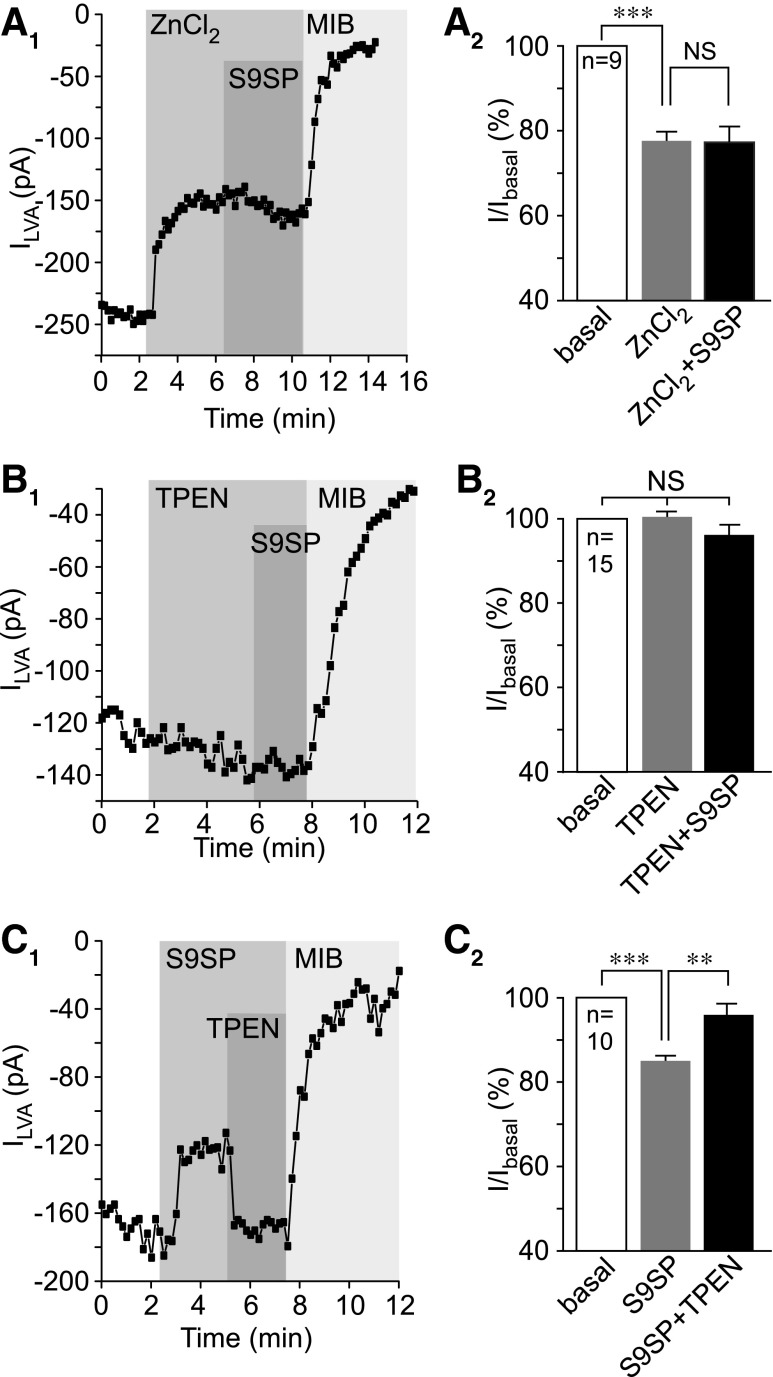
**Extracellular zinc mimics SP action on T-type Ca^2+^ channels. (A)** Extracellular ZnCl_2_ inhibited LVA Ca^2+^ currents in DRG neurons. **(A_1_)** Example time course of the effect of ZnCl_2_ (750 n*M*), S9SP (1 μ*M*), and MIB (3 μ*M*) on the LVA Ca^2+^ current recorded form small-diameter DRG neurons using whole-cell patch clamp. Periods of drug application are indicated by the *vertical gray bars*; **(A_2_)** summarizes the effects. **(B)** Zinc chelator TPEN (10 μ*M*) prevents SP-mediated LVA current inhibition in DRG neurons. **(B_1_)** Example time course of the effect of TPEN (10 μ*M*), S9SP (1 μ*M*), and MIB (3 μ*M*) on the LVA current; **(B_2_)** summarizes the effects; layout and labeling are similar to **(A)**. **(C)** TPEN reverses the SP-induced LVA current inhibition in DRG neurons. **(C_1_)** Example time course of the effect of S9SP (1 μ*M*), TPEN (10 μ*M*), and MIB (3 μ*M*) on the LVA current; **(C_2_)** summarizes the effects; layout and labeling are similar to **(A, B)**. *Asterisks* indicate significant difference from the group indicated by the *line connector* with **p < 0.01 or ***p < 0.001 (paired *t*-test). In all bar charts, data are shown as mean ± SEM. TPEN, N,N,N′,N′*-*Tetrakis(2-pyridylmethyl)ethylenediamine.

Application of 750 n*M* ZnCl_2_ caused inhibition of LVA current in DRG neurons by ∼25% (*p* < 0.001; *n* = 9; Fig. 3A), but when S9SP was applied in the presence of zinc, it had no further inhibitory effect (Fig. 4A). Furthermore, S9SP did not produce any effect in the presence of the zinc chelator N,N,N′,N′*-*Tetrakis(2-pyridylmethyl)ethylenediamine (TPEN; 10 μ*M*; Fig. 4B and Supplementary Fig. S4). S9SP-induced inhibition was completely reversed by bath application of TPEN (still in the presence of S9SP, Fig. 4C and Supplementary Fig. S4). Similar to DRG neurons, SP- and H_2_O_2_-induced inhibitions of recombinant Ca_V_3.2 were prevented by TPEN (Supplementary Fig. S1C, D).

To accept a hypothesis that SP promotes T-type channel inhibition by ambient zinc and that this effect is mediated by a redox-dependent mechanism, two questions need to be addressed. (i) Is there enough ambient zinc to mediate the observed effects (as all our solutions are nominally zinc free, unless stated otherwise)? (ii) Does SP induce any changes to extracellular or intracellular zinc levels in DRG neurons?

**FIG. 5.**
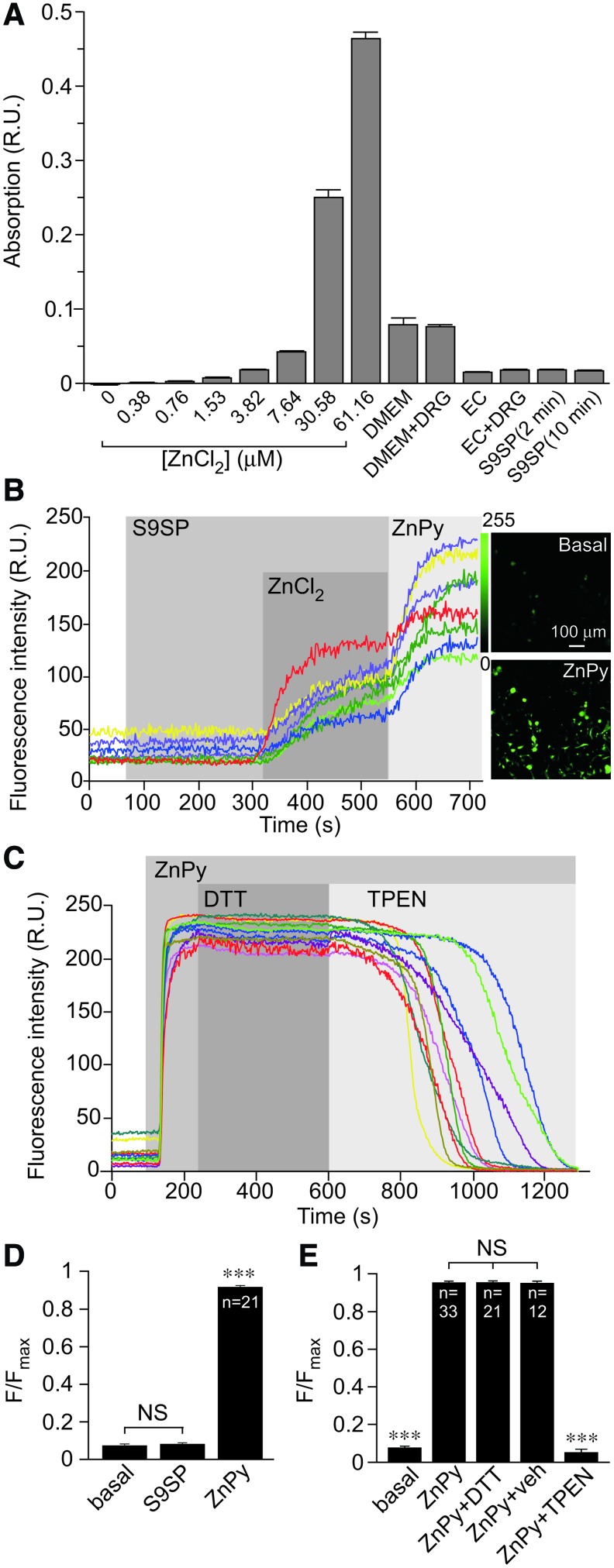
**SP does not change extracellular or intracellular zinc levels in DRG cultures. (A)** Nominally zinc-free media contains micromolar levels of zinc. Atomic absorption spectroscopy measurements of total zinc in experimental media (as indicated); *left* eight bars correspond to the standard solutions of ZnCl_2_ in de-ionized water. Bars in the *middle* correspond to the levels of zinc in experimental media: DMEM is a DRG culture medium tested either on its own or collected from a DRG culture plate (“DMEM” and “DMEM+DRG”); EC is an extracellular solution used in patch-clamp and imaging experiments tested either on its own or collected from a DRG culture plate (“EC” and “EC+DRG”). Two bars on the *right* correspond to the experiments where zinc content has been analyzed in the EC of DRG cultures treated with 1 μ*M* S9SP for 2 or 10 min (as indicated). **(B)** SP does not produce detectable rises in intracellular zinc levels in DRG neurons. Fluorescence imaging of cultured small-diameter DRG neurons loaded with zinc fluorophore, FluoZin^™^-3AM. Example time course of the effect of S9SP (1 μ*M*), ZnCl_2_ (1.5 m*M*) and zinc ionophore, ZnPy (10 μ*M*) on FluoZin-3 fluorescence. Corresponding images of the culture under basal conditions and after the application of ZnPy (as indicated) are shown on the *right*. **(C)** Increase in FluoZin-3 fluorescence induced by 10 μ*M* ZnPy is completely reversed by 20 μ*M* zinc chelator, TPEN but is unaffected by 1 m*M* DTT. **(D, E)** Summarize the experiments presented in **(B, C),** respectively. **(D)** *Asterisks* indicate significant difference from the basal fluorescence with ***p < 0.001 (paired *t*-test); **(E)** *Asterisks* indicate significant difference from the fluorescence in the presence of ZnPy with ***p < 0.001 (paired *t*-test). In all bar charts, data are shown as mean ± SEM. DMEM, Dulbecco's modified Eagle medium; ZnPy, zinc pyrithione. To see this illustration in color, the reader is referred to the web version of this article at www.liebertonline.com/ars

First, we measured the zinc content of extracellular solutions and media using the atomic absorption spectroscopy (AAS; Fig. 5A). Both the DRG culture medium and the extracellular bath solution contained micromolar concentrations of zinc (∼10 μ*M* in Dulbecco's modified Eagle medium [DMEM] and ∼5 μ*M* in extracellular bath solution), which were similar to the reported plasma zinc levels in humans (44). There were no detectable changes in zinc content in the extracellular media after up to 10 min incubation of DRG cultures with S9SP (Fig. 5A). Notably, AAS detects total zinc content and the free zinc is likely to be significantly lower than the 5–10 μ*M*; this can explain the significant inhibition of LVA current observed in the extracellular solution supplemented with 750 n*M* ZnCl_2_ (Fig. 4A).

Next, we measured intracellular zinc levels using confocal fluorescence microscopy of DRG cultures loaded with the zinc indicator FluoZin^™^-3AM. Application of S9SP did not produce detectable changes in intracellular zinc levels; however, large responses were recorded in response to 1.5 m*M* ZnCl_2_ or zinc ionophore zinc pyrithione (ZnPy; 10 μ*M*; Fig. 5B, D). In a separate experiment, we detected significant intracellular zinc transients in response to acidification (pH 5.5; Supplementary Fig. S5). Acidification has been previously shown to release intracellular zinc from cytosolic zinc-cysteine complexes (28) and, thus, acidification-induced zinc transients are likely to represent intracellular zinc release.

We then tested whether DTT can chelate zinc, as has been suggested (30, 48). We first loaded DRG cultures with zinc by the application of ZnPy and then applied, in sequence, 1 m*M* DTT and 20 μ*M* TPEN (still in the presence of ZnPy; Fig. 5C, E). Cell-permeable (19) DTT did not alter the elevated zinc levels, whereas TPEN completely reversed the increase in fluorescence produced by ZnPy (Fig. 5C, E). This suggests that TPEN effectively competed for zinc binding with the zinc-sensitive dye at a molar ratio of 2:1, whereas DTT was unable to produce such an effect even at a molar ratio of 200:1. The experiments presented in Figure 5 and Supplementary Figure S5 established that (i) ambient zinc is present in nominally zinc-free solutions and media at levels that are sufficient to affect Ca_V_3.2 activity (27, 66); (ii) we could not detect significant changes in intracellular or extracellular zinc levels in DRG neurons in response to SP; and (iii) we could not detect zinc chelator activity of DTT. Although this last observation cannot rule out zinc binding by DTT, in principle, it argues against zinc chelation by DTT as the reason underlying the reversibility of the SP effect on T-type channels by DTT in our experiments. Instead, we hypothesize that oxidation increases and reduction decreases channel sensitivity to zinc. We further suggest that SP induces T-type channel inhibition in DRG neurons by producing ROS-mediated modification of T-type channel protein, which, in turn, alters the channel sensitivity to zinc.

**FIG. 6.**
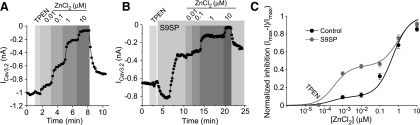
**SP increases zinc-mediated inhibition of Ca_V_3.2 that is mediated by a high-affinity binding site. (A)** Exemplary patch-clamp recording from HEK293 cell co-transfected with Ca_V_3.2 and NK1 showing concentration dependency of the effect of extracellular ZnCl_2_ on Ca_V_3.2 currents. TPEN (10 μM) was applied at the beginning of the experiment to record Ca_V_3.2 current amplitude at a near-zero extracellular zinc concentration. Increasing concentrations of ZnCl_2_ were applied during the periods indicated by *vertical gray bars*. **(B)** Experiment similar to **(A)** but increasing ZnCl_2_ concentrations were applied after application of 1 μ*M* S9SP; 1 μ*M* S9SP was present in all ZnCl_2_ solutions. **(C)**. The data from 6–12 experiments as in **(A, B)** were fit to two-component logistic function (see Materials and Methods section). The solution containing 10 μ*M* TPEN and no added ZnCl_2_ was assigned a free zinc concentration of 0.05 n*M*. In control conditions, the IC_50(1)_ was 0.25 ± 1.4 n*M* and the IC_50(2)_ was 495 ± 326 n*M*. After the S9SP treatment, the IC_50(1)_ was 0.35 ± 0.25 n*M* and the IC_50(2)_ was 923 ± 582 n*M*. **(C)** Data are shown as mean ± SEM. HEK, human embryonic kidney.

To directly test whether SP treatment changes zinc sensitivity of Ca_V_3.2, we investigated concentration dependency of zinc effect on the recombinant Ca_V_3.2 overexpressed in HEK293 cells together with NK1 receptors with and without S9SP treatment. We applied 10 μ*M* TPEN in the nominally zinc-free bath solution at the beginning of each of such experiments to achieve near-zero extracellular free zinc levels and then applied increasing concentrations of ZnCl_2_ (now without TPEN; Fig. 6A, B).

The concentration dependency was best fit with the two-component logistic function (Fig. 6C; see Materials and Methods section). The data obtained in control conditions could be fit well with the one-component function as well [in agreement with previous reports (27, 66); not shown], as the inhibition at low nanomolar zinc concentrations was small. However, a far better fit was achieved with the two-component logistic function, which clearly separated two zinc effects (Fig. 6C). Such bi-phasic concentration dependence is likely to indicate that Ca_V_3.2 uniquely possesses high-affinity (sub-micromolar) and low-affinity (micromolar) zinc binding sites. The latter is likely to be shared with other Ca_V_3 subunits and other types of VGCC channels, as these are also inhibited by micromolar (but not nanomolar) zinc levels (6, 27, 66).

S9SP treatment strongly increased the efficacy of zinc-mediated inhibition *via* the high-affinity mechanism while having no effect on the inhibition at higher concentrations (Fig. 6C). The maximal inhibition at the high-affinity site in the control and S9SP-treated cells was 9.9% ± 11.3% and 44.1% ± 5.1%, respectively; the IC_50_ values for high- and low-affinity sites did not change significantly. We estimated that free zinc concentrations in our solutions are in the low nanomolar range, since S9SP inhibited Ca_V_3.2 in nominally zinc-free solutions by ∼40% (Supplementary Fig. S1A and Fig. 6); however, TPEN produced only marginal augmentation of Ca_V_3.2 currents in control conditions (Supplementary Fig. S1C, D and Fig. 6A, B). These experiments also explain why application of 750 n*M* extracellular zinc abolished the S9SP effect (Fig. 4A), as at concentrations higher than 100 n*M* the difference in zinc-induced inhibition between control and S9SP-treated cells started to disappear (Fig. 6C). The presence of low-nanomolar free zinc in nominally zinc-free solutions introduces a slight systematic error to the concentration-dependency measurements; however, we believe that the overall conclusion that SP treatment enhances inhibition of Ca_V_3.2 by zinc acting at its high-affinity site is valid.

**FIG. 7.**
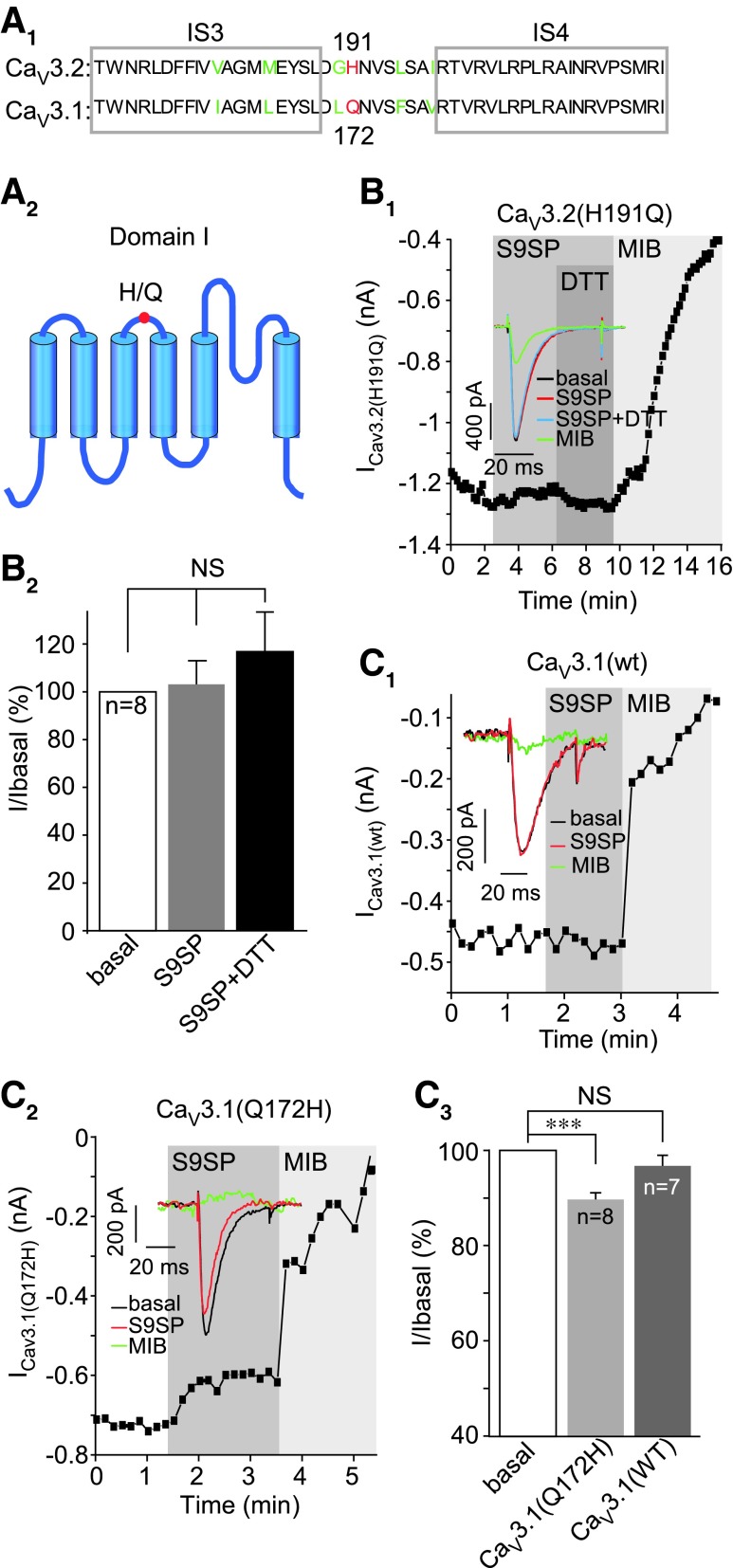
**High-affinity zinc binding site is necessary for Ca_V_3 channel sensitivity to SP. (A_1_)** Depicts the amino-acid sequence alignment of the transmembrane segments S3 and S4 of domain I (with extracellular linker) of Ca_V_3.1 and Ca_V_3.2; non-conserved H191 of Ca_V_3.2 is highlighted in *red*. **(A_2_)** Simplified schematic of domain I of Ca_V_3 channels. **(B)** H191Q substitution in Ca_V_3.2 renders the channel insensitive to SP inhibition. **(B_1_)** Example time course of the effect of S9SP (1 μ*M*), DTT (1 m*M*), and MIB (3 μ*M*) on the Ca_V_3.2(H191Q) mutant recorded in HEK293 cell co-transfected with NK1 receptor; *inset* shows example current traces. **(B_2_)** Shows summary of the effects. **(C)** Q172H substitution in Ca_V_3.1 renders the channel sensitive to SP inhibition. **(C_1_, C_2_)** Example time courses of the effects of S9SP (1 μ*M*) and MIB (3 μ*M*) on the wtCa_V_3.1 **(C_1_)** and Ca_V_3.1(Q172H) mutant **(C_2_)** recorded in HEK293 cell co-transfected with NK1 receptor. *Insets* show example current traces; **(C_3_)** shows summary of the effects. The number of experiments is shown within the bars in **(B_2_, C_3_)**. *Asterisks* indicate significant difference from the group indicated by the *line connector* with ***p < 0.001 (paired *t*-test). In all bar charts, data are shown as mean ± SEM. To see this illustration in color, the reader is referred to the web version of this article at www.liebertonline.com/ars

The unique high-affinity metal-binding site of Ca_V_3.2 has been elucidated (27): It is composed of an Asp-Gly-His motif in the extracellular S3–S4 linker of the domain I and a separate Asp in the S1–S2 linker of the same domain (Fig. 7A_1,_ A_2_); this site is also critical for channel modulation by redox agents (26, 27, 47, 48). His191 plays a central role in the zinc-mediated channel inhibition. This residue is absent in Ca_V_3.1 and Ca_V_3.3; accordingly, these channels are far less sensitive to zinc.

To test whether SP-mediated Ca_V_3.2 channel inhibition requires this high-affinity zinc-binding site, we tested the SP sensitivity of two reciprocal mutants: Ca_V_3.2 with H191 substituted by glutamine (Ca_V_3.2H191Q) and Ca_V_3.1 with H-to-Q substitution at the homologous position 172 (Ca_V_3.1Q172H; Fig. 7A_1_, A_2_). Notably, Ca_V_3.2H191Q displayed no sensitivity to SP (Fig. 7B); current amplitude in the presence of S9SP was 103.1% ± 9.8% of basal value (*n* = 8) *versus* 69.8% ± 6.8% for the wtCa_V_3.2 (*p* < 0.001; *n* = 13; Supplementary Fig. S1A). Similarly insensitive to S9SP was the wild-type Ca_V_3.1 (Fig. 7C_1_); whereas Ca_V_3.1Q172H mutant, in contrast, displayed small but significant inhibition by S9SP (10.4% ± 1.6%; *p* < 0.05; *n* = 8; Fig. 7C_2_, C_3_). In accord with previous reports, Q172H substitution in Ca_V_3.1 only partially restored channel sensitivity to sub-millimolar zinc, as the entire binding site is required for the full effect (27).

### Effects of novel T-type Ca^2+^ channel modulators on sensory neuron excitability

**FIG. 8.**
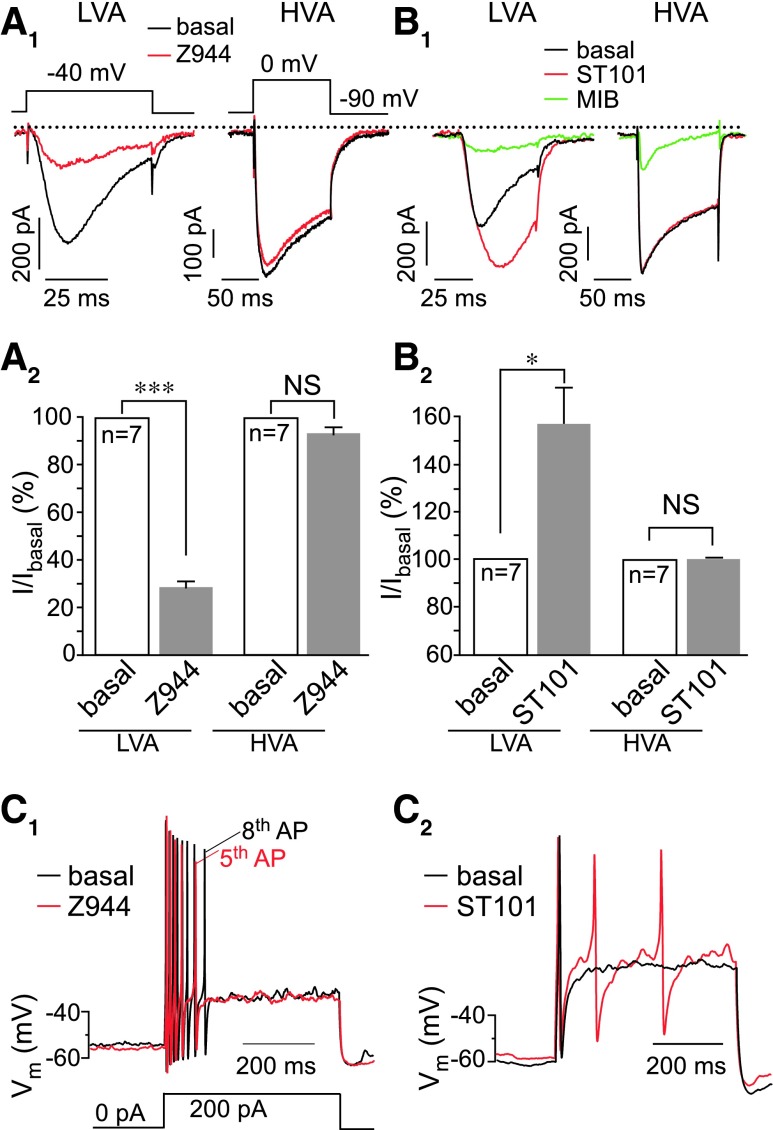
**Modulation of T-type channel activity in DRG neurons reveals the channel's role in control over the excitability. (A)** Z944 potently inhibits LVA but not HVA Ca^2+^ currents in DRG neurons. **(A_1_)** Shows example whole-cell patch clamp recordings, and **(A_2_)** shows summarized data for the effects of 1 μ*M* Z944 on LVA (*left*) and HVA (*right*) Ca^2+^ currents; voltage protocols are depicted above the traces. **(B)** ST101 potently augments LVA but not HVA Ca^2+^ currents in DRG neurons; layout and labeling are similar to **(A_1_, A_2_)**; to inhibit HVA current, MIB was used at 10 μ*M*. **(A, B)** HVA currents were recorded from neurons lacking the LVA current. *p < 0.05, ***p < 0.001; paired *t*-test. **(C_1_, C_2_)** Effect of Z944 **(C_1_)** and a T-type channel activator, ST101 **(C_2_)** on small DRG neuron excitability recorded in perforated patch-clamp current mode. Current injection protocol is depicted beneath the traces in **(C)**. Extended analysis of the ST101 and Z944 effects on excitability is given in Supplementary Figure S7. **(A, B)** Data are shown as mean ± SEM. HVA, high-voltage activated; ST101, spiro[imidazo[1,2-a]pyridine-3,2-indan]-2(3H)-one. To see this illustration in color, the reader is referred to the web version of this article at www.liebertonline.com/ars

The T-type Ca^2+^ current is an important regulator of sensory neuron excitability (10, 17, 18, 20, 23, 24, 51, 71, 75). To evaluate the contribution of T-type Ca^2+^ channel modulation to the effects of SP on sensory neuron excitability both *in vitro* and *in vivo*, we used recently developed specific and selective T-type Ca^2+^ channel modulators: spiro[imidazo[1,2-a]pyridine-3,2-indan]-2(3H)-one (ST101), a highly active Ca_V_3 potentiator (13, 45) with an EC_50_ in sub-nanomolar range, and Z944, a selective Ca_V_3 inhibitor with IC_50_ ∼50 n*M* (67). Z944 (1 μ*M*) inhibited LVA current in DRG neurons by 71.4% ± 2.7% (*p* < 0.001; *n* = 7), without a noticeable effect on HVA current (Fig. 8A); at this concentration, Z944 produced no significant effect on L- and N-type VGCCs, Nav1.5 and hERG channels (67).

ST101 (1 n*M*) enhanced peak LVA current in DRG neurons by 50.6% ± 14.1% (*p* < 0.01; *n* = 7), also without an effect on HVA current (Fig. 8B); at 1 n*M*, ST101 had no effect on delayed-rectifier K^+^ current and voltage-gated Na^+^ current in DRG neurons (Supplementary Fig. S6). Although several selective blockers of T-type Ca^2+^ channels were reported (10, 17), selective T-type channel potentiators were not available until recently.

We next tested the effect of Z944 and ST101 on DRG neuron excitability using the perforated current clamp technique. Z944 decreased (Fig. 8C_1_) and ST101 increased (Fig. 8C_2_) small DRG neuron excitability; these effects are further analyzed in Supplementary Figure S7: Z944 significantly increased the rheobase, decreased action potential (AP) firing, and hyperpolarized small-diameter DRG neurons without significantly affecting AP amplitude or duration. In contrast, ST101 significantly increased AP firing and depolarized E_rest_; AP amplitude or duration was not affected (Supplementary Fig. S7A–C).

S9SP produced a pattern of effects similar to that of Z944 (Supplementary Fig. S7A–C), although only the effect on rheobase (Supplementary Fig. S7A) reached significance. Of note, mean values and the statistical significance of the effects shown in Supplementary Figure S7 are underestimated, since not all DRG neurons express all the necessary components of the signaling cascade. Thus, ∼40% of small DRG neurons in our cultures express LVA currents; ∼40% to 50% of small neurons express functional NK1 receptors [(33), present study]; and ∼70% of LVA-expressing neurons respond to S9SP. Therefore, in each series of experiments, there was a subpopulation of “unresponsive” neurons.

We further tested whether SP or a T-type Ca^2+^ channel blocker would attenuate the excitatory effect of the pro-algesic peptide bradykinin (BK) (36, 52). BK (250 n*M*) significantly reduced the rheobase, increased the AP number, and depolarized the E_rest_ (Supplementary Fig. S7A–C). When co-applied with BK, both S9SP and Z944 completely reversed the effects of BK on the rheobase and the AP number (Supplementary Fig. S7A, B). The BK-induced depolarization in the presence of either S9SP or Z944 was more variable (Supplementary Fig. S7C), perhaps reflecting the complexity of the depolarizing effects of BK (25, 36, 52), but in both cases the effect of BK was no longer statistically significant.

To confirm that the effects of Z944 and ST101 on sensory neuron excitability are, indeed, mediated by T-type channels, we repeated some of the current clamp experiments in DRG neurons in which Ca_V_3.2 expression has been downregulated with siRNA against *Cacna1h*. The procedure (see Materials and Methods section) reduced the *Cacna1h* transcript levels by ∼50% while having no effect on the housekeeping transcript (*Gapdh*; Supplementary Fig. S8A, B). In the *Cacna1h* knocked-down neurons, neither Z994 nor ST101 produced any significant effect on E_rest_, AP number, or rheobase (Supplementary Fig. S8C–E); whereas in scrambled oligonucleotide-transfected neurons, both drugs were still effective (*cf.* Supplementary Figs. S7 and S8C–E).

Taken together, the experiments reported in Figure 8 and Supplementary Figures S7 and S8 suggest that (i) T-type channels modulate excitability of many small-diameter DRG neurons; (ii) acute inhibition of T-type channels produces an anti-excitatory effect that can partially offset the excitatory action of BK; (iii) SP produces an anti-excitatory effect that is qualitatively similar to that produced by a T-type channel blocker; and (iv) effects of Z944 and ST101 on DRG neuron excitability are, indeed, mediated by T-type Ca^2+^ channels.

### T-type Ca^2+^ channel inhibition contributes to the peripheral anti-nociceptive effect of SP *in vivo*

**FIG. 9.**
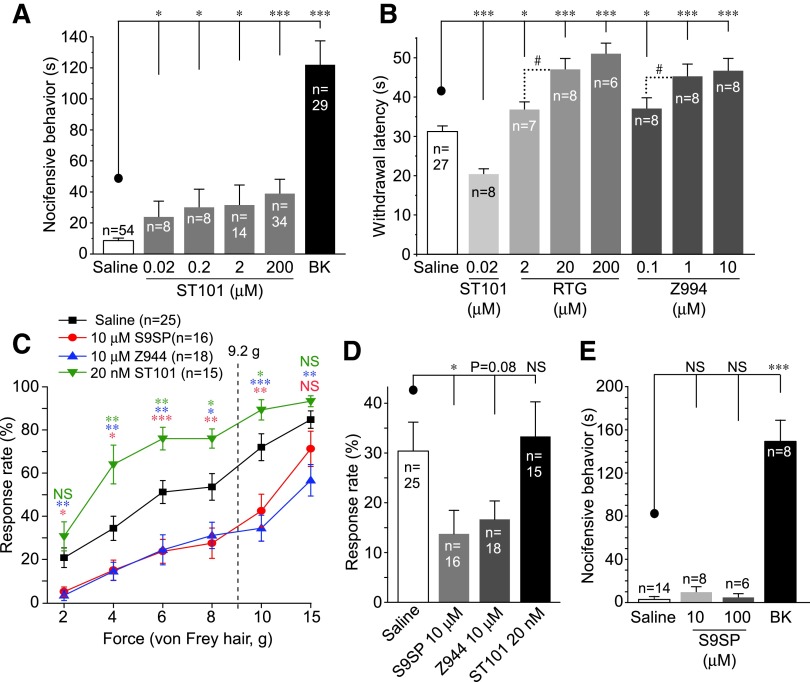
**T-type Ca^2+^ channel contributes to the peripheral analgesic effect of SP. (A)** Plantar hind-paw injection of ST101 (50 μl; 0.001–10 nmol/site) and BK (50 μl; 10 nmol/site) induced nocifensive behavior in rats. Nocifensive behavior (time spent licking, flinching, and biting the paw) was quantified over the period of 30 min. Extended analysis of the data is given in Supplementary Figure S9. **(B)** Effects of plantar hind-paw injections of ST101 (0.001 nmol/site), M channel opener, RTG (50 μl; 0.1–10 nmol/site), Z944 (50 μl; 0.005–0.5 nmol/site), or saline on the paw withdrawal latency on exposure to noxious heat (Hargreaves test). **(C)** Effects of plantar hind-paw injections of ST101 (50 μl; 0.001 nmol/site), Z944 (50 μl; 0.5 nmol/site), S9SP (50 μl; 0.5 nmol/site), or saline on the response rate on application of von Frey filaments of varying bending force to the plantar surface of the hind paw. *Vertical dotted line* indicates 60% withdrawal threshold of saline-injected rats. **(D)** Effects of plantar hind-paw injections of ST101 (50 μl; 0.001 nmol/site), Z944 (50 μl; 0.5 nmol/site), S9SP (50 μl; 0.5 nmol/site), or saline on the sensitivity to gentle touch in cotton-swab test. **(E)** Plantar hind-paw injection of S9SP (50 μl; 0.5–5 nmol/site) failed to induce nocifensive behavior in rats (in contrast to BK). ^#^, *, **, ***Significantly different from the control group **(C)** or from the group indicated by the *line connector* (all other panels) with *p* < 0.05, *p* < 0.01, or *p* < 0.001; Kruskal–Wallis ANOVA and Mann–Whitney test. All data are shown as mean ± SEM. ANOVA, analysis of variance; BK, bradykinin; RTG, retigabine. To see this illustration in color, the reader is referred to the web version of this article at www.liebertonline.com/ars

We next investigated the influence of T-type channels on peripheral sensory afferents *in vivo*. First, we tested whether T-type channel augmentation is, indeed, pro-nociceptive. An injection of ST101 (50 μl; 0.001–10 nmol/site) into the rat hind paw induced a moderate but significant protective effect (“nocifensive” behavior, see Materials and Methods section) that was indicative of moderate pain. The pro-algesic effect of ST101 was smaller than that induced by BK (50 μl; 10 nmol/site), but it was significantly stronger than that of saline (Fig. 9A).

Time courses of the nociceptive effects of BK and ST101 are shown in Supplementary Figure S9. The nocifensive behavior induced by BK usually tailed out after 15 min post-injection (Supplementary Fig. S9A); whereas the effects of ST101 showed a distinct pattern, with lower concentrations causing a delayed response and higher doses producing more immediate and shorter effects (Supplementary Fig. S9B). Such different kinetic patterns may indicate that excitations produced by BK and ST101 have distinct mechanisms.

We then evaluated the effects of Z944, ST101, and an M-type K^+^ channel activator, retigabine (RTG), on animal sensitivity to noxious heat using the Hargreaves’ method. RTG has well-known peripheral analgesic activity (13, 21, 36, 50), and M-channel potentiation also contributes to the endogenous anti-nociceptive effect of SP (33). A peripheral hind-paw injection of Z944 (50 μl; 0.005–0.5 nmol/site) significantly increased paw withdrawal latency on radiant heat exposure, suggesting decreased heat sensitivity (Fig. 9B). RTG (50 μl; 0.1–10 nmol/site) produced a similar effect. In contrast, ST101 significantly reduced withdrawal latency, indicating increased heat sensitivity (Fig. 9B); time courses of these effects of Z944 and RTG are given in Supplementary Figure S9C.

Ca_V_3.2 is expressed in subpopulations of Aδ and C low-threshold mechanoreceptors (LTMRs) and plays a role in tuning sensitivity to light touch (18). Thus, we tested the effects of local application of Z944, ST101, and S9SP on a range of mechanical stimulations.

In von Frey test (see Materials and Methods section), saline-injected rats had a 60% withdrawal threshold at 9.2 ± 1.0 g (*n* = 25). Injections of Z944 (50 μl; 0.5 nmol/site) and S9SP (50 μl; 0.5 nmol/site) significantly impaired their ability to respond to stimulation, ranging from innocuous to noxious; Z944 was more efficacious in the noxious range (Fig. 9C). In contrast, an injection of ST101 (50 μl; 0.001 nmol/site) greatly increased response rates (Fig. 9C). The cotton swab assay (see Materials and Methods section) also showed significantly reduced sensitivity to light touch after the S9SP injection (50 μl; 0.5 nmol/site); Z944 (50 μl; 0.5 nmol/site) produced a similar effect (Fig. 9D). An injection of ST101, however, was ineffective in this type of test.

We then tested the contribution of T-type channel modulation to the peripheral effect of S9SP. Consistent with previous reports (33), a local hind-paw injection of S9SP (0.5–5 nmol/site) did not produce measurable nocifensive behavior; whereas BK induced a strong nocifensive response (Fig. 9E).

**FIG. 10.**
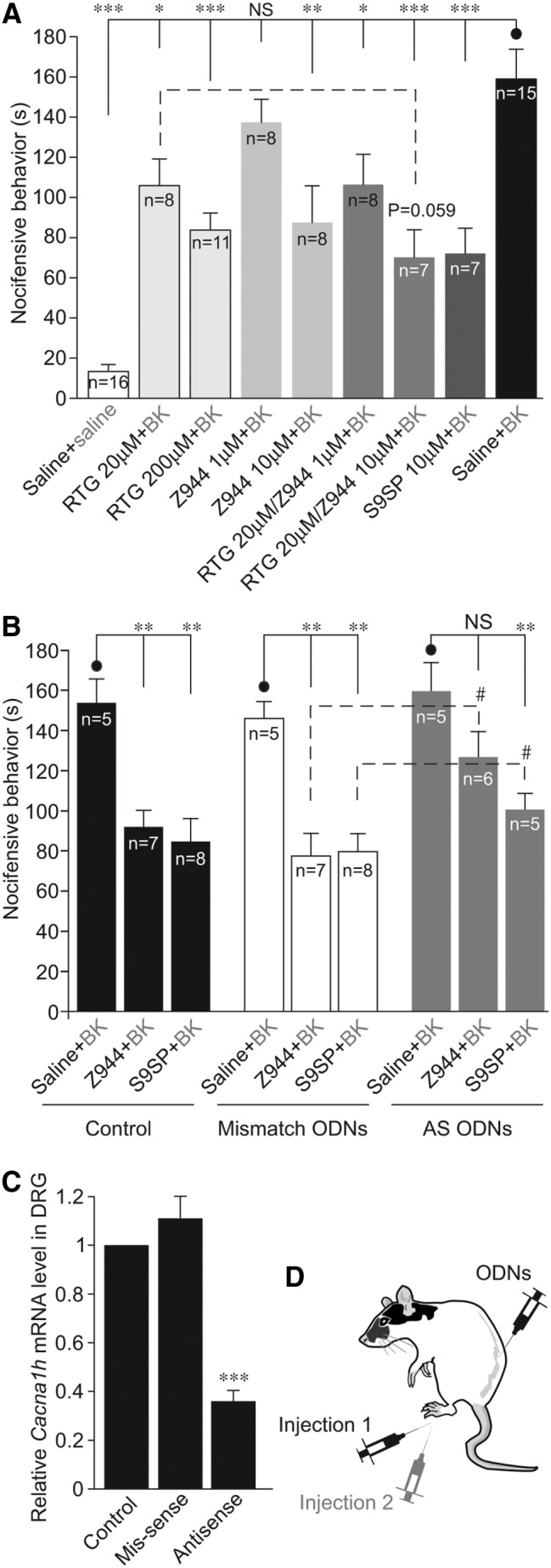
**NK1 receptor-mediated T-type Ca^2+^ channel inhibition is a likely contributor to the anti-nociceptive effect of SP. (A)** Plantar hind-paw injections of S9SP (50 μl; 0.5 nmol/site), RTG (50 μl; 1–10 nmol/site), Z944 (50 μl; 0.05–0.5 nmol/site) or a combination of RTG+Z944 solutions (at concentrations indicated) were administered 5 min before the injection of BK (10 nmol/site) and mixed together with the pre-injection drug or drug mixture in 50 μl saline [schematic of the double-injection protocol is shown in **(D)**]. The number of animals in each group is shown within the bars. **(B)** Intrathecal knock-down of Ca_V_3.2 in lumbar DRG reduces anti-nociceptive effect of Z994 and S9SP. Animals with intrathecal injection of saline, Ca_V_3.2 AS ODNs, or non-targeting “mismatch” ODNs were investigated. Hind-paw injections of S9SP (50 μl; 0.5 nmol/site) or Z944 (50 μl; 0.5 nmol/site) were administered 5 min before the injection of BK (10 nmol/site), similar to experiments shown in **(A)**. **(C)** Quantification of knock-down efficiency; the analysis was performed on the second day after the last intrathecal injection. The Ca_V_3.2 transcript levels in lumbar DRGs of AS ODN- and mismatch ODN-injected animals were normalized to those of saline-injected animals in the same experimental run; one to three animals per run, three independent runs. *, **, ***Significantly different from the group indicated by the line connector with *p* < 0.05, *p* < 0.01, or *p* < 0.001; Kruskal–Wallis ANOVA and Mann–Whitney test. All data are shown as mean ± SEM. AS, antisense; ODNs, oligodeoxynucleotides.

We then pre-injected RTG, Z944, or S9SP into the rat hind paw 5 min before the BK injection (together with the same pre-injection drug; Fig. 10A, D). RTG served as a positive control, as it was shown to offset excitatory effects of BK and to reduce the BK-induced nociception (33, 34, 36). Pre-injections of both RTG (50 μl; 1–10 nmol/site) and Z944 (at 0.5 nmol/site but not at 0.05 nmol/site) significantly attenuated the BK-induced nociception. A pre-injection of S9SP (0.5 nmol/site) also strongly suppressed the BK-induced nocifensive behavior (Fig. 10A). Importantly, a mixture of RTG (1 nmol/site) and Z944 (0.5 nmol/site) was as efficacious as S9SP in reducing BK-induced nocifensive behavior and showed a tendency to be more efficacious than RTG or Z944 injected alone (*p* = 0.059 *vs.* RTG alone; Fig. 10A); this finding is consistent with a hypothesis that M-channel augmentation and T-type channel inhibition can be cumulative components of peripheral anti-nociceptive effects of SP.

Finally, we performed *in vivo* knock-down of Ca_V_3.2 in DRG neurons by an intrathecal injection of antisense (AS) phosphorodiester oligodeoxynucleotides (ODNs) using the protocol that had been successfully used earlier for the same purpose [(3, 41); Materials and Methods section]. In saline- and mismatch ODN-injected animals, both Z944 (0.5 nmol/site) and S9SP (0.5 nmol/site) still significantly attenuated nocifensive behavior produced by the hind-paw BK injection. However, in Ca_V_3.2 AS ODN-injected animals, the nocifensive behavior produced by both Z944 and S9SP was significantly reduced (Fig. 10B). The levels of Ca_V_3.2 transcript in lumbar DRGs of Ca_V_3.2 AS ODN-injected animals were reduced by ∼70% as compared with the saline-injected controls and mismatch ODN-injected animals (Fig. 10C).

## Discussion

Here, we described a hitherto unknown endogenous signaling pathway linking NK1 receptors with Ca_V_3.2 channel activity and demonstrated that this pathway is involved in the paradoxical peripheral anti-nociceptive effect of SP. T-type Ca^2+^ channels are sensitive to redox modulation; oxidizing agents or ROS inhibit channel activity (47, 65), whereas reducing agents such as L-cysteine or DTT reverse such inhibition and can augment channel activity above the basal level in some cases [(46, 65); Fig. 3 and Supplementary Fig. S1].

SP induces ROS release in immune (64) and epithelial (60) cells and in DRG neurons (33). Accordingly, SP-induced inhibition of T-type Ca^2+^ channels observed in this study displayed the following features: (i) It was fully reversed or prevented by DTT (Fig. 3); (ii) mimicked by exogenously applied H_2_O_2_ (Fig. 3 and Supplementary Fig. S1); and (iii) stimulated by endogenous ROS release (Fig. 3E).

The mechanism of the redox sensitivity of T-type Ca^2+^ channels is not entirely understood, but it requires the extracellular His191 in Ca_V_3.2; the other Ca_V_3 isoforms do not have a corresponding histidine and are largely redox insensitive (47). The same H191 mediates Ca_V_3.2 inhibition by sub-micromolar concentrations of zinc (26, 27). H191 is a general metal-binding site, and it was hypothesized that it could participate in the metal-catalyzed oxidation (MCO) reaction accelerated by transition metals (47, 61).

It is also possible that an oxidative modification (possibly but not necessarily at H191) can promote zinc binding or potentiate its action on the channel, thus sensitizing it to ambient concentrations of this trace metal. The following observations are in favor of this latter mechanism. (i) SP induced a large increase in zinc-mediated inhibition at the high-affinity site in Ca_V_3.2 (Fig. 6). (ii) A mutation of H191 in Ca_V_3.2 rendered the channels insensitive to SP (Fig. 7). (iii) Ca_V_3.1 is insensitive to SP but insertion of His into the position equivalent to H191 in Ca_V_3.2 renders the Ca_V_3.1 channel sensitive to SP (Fig. 7). (iv) The zinc chelator TPEN prevents or reverses SP-induced T-type Ca^2+^ current inhibition (Fig. 4 and Supplementary Fig. S1). (v) Extracellular zinc inhibits LVA currents in DRG neurons, an effect that is similar in amplitude to that produced by SP (Fig. 4). (vi) Extracellular zinc occludes any further inhibitory actions of SP (Fig. 4). (vii) We could not detect any changes in intracellular or extracellular zinc levels induced by SP (Fig. 5 and Supplementary Fig. S5). (viii) Nominally zinc-free media contain low-nanomolar concentrations of free zinc, which are sufficient to affect channel activity when the channel is sensitized by SP (Fig. 6); however, these levels are too low to significantly inhibit T-type channels in their tonic state. The last observation would explain why TPEN produced only a marginal effect on T-type currents under basal conditions (Figs. 4B and 6B and Supplementary Fig. S1C, D).

Despite previous suggestions (30, 48), we could not detect zinc chelation by DTT under our experimental conditions (Fig. 5). Therefore, we suggest that the reversal of the SP-mediated T-type channel inhibition by DTT is due to the reducing activity of the latter.

Interestingly, there are three extracellular cysteines (residues that can be easily modified by redox mechanisms) in the IS1-IS2 linker of Ca_V_3.2, a domain that contributes to the high-affinity zinc-binding site (27). We hypothesize that cumulative oxidation of such extracellular cysteines may result in a graded increase in channel sensitivity to zinc. In such a scenario, Ca_V_3.2 tonically exists in a partially “oxidized” state with intermediate zinc sensitivity, which is, however, too low for inhibition by ambient zinc. This could be why in our hands TPEN or DTT had only marginal effects on channel activity under control conditions.

The partially oxidized state can then be converted to a fully oxidized state, with the highest zinc sensitivity by SP or ROS, or to a fully reduced state, with the lowest zinc sensitivity by DTT. This could account for the augmentation of tonic Ca_V_3.2 currents by DTT (65) and TPEN (15) that are observed in some labs, as different experimental conditions may result in different “tonic” redox states of the channel (*e.g.*, DTT or TPEN would potentiate fully oxidized channels). This hypothesis requires further verification.

The levels of NK receptor expression in peripheral sensory neurons are reportedly low. Thus, one immunohistochemical study found little evidence for NK receptor immunoreactivity in rat sensory neurons (5); whereas recent RNAseq experiments found very low levels of NK receptor transcripts in mouse DRG (39, 68), although the levels were higher in human DRG (16). However, in both mouse (39) and human (16) DRG, the NK receptor transcript levels were comparable (or even higher) to those of the brain or lung, tissues where prominent SP signaling is well characterized. Moreover, the DRG NK receptor levels are comparable to those of BK B_2_ receptors (16, 39), which exert robust effects in DRG neurons [reviewed in Petho and Reeh (52)].

Perhaps low transcript levels and expression in only specific cell populations are sufficient for functional activity of such G-protein-coupled receptors. In support of this notion, *in vitro* and, particularly, *in vivo* evidence presented here and elsewhere (32, 33, 73) clearly demonstrate the presence of functional SP signaling in DRG. In addition, NK1 receptor immunoreactivity was found throughout DRG neuron somata and often co-localized with that of Ca_V_3.2, especially in small-diameter neurons (Supplementary Fig. S2).

There is also some discrepancy regarding cellular effects of SP in cultured DRG neurons. Thus, some potentially excitatory effects, including depolarization (1, 74), potentiation of Nav1.8 Na^+^ channels (7), L- and N-type Ca^2+^ channels (58), and sensitization of TRPV1 (33, 57, 73), were reported. However, there is no acute pain produced by local SP injections [(33); present study]. Application of SP to human skin by dermal microdialysis produces no painful sensation or primary afferent excitation (72). Thus, at present, the excitatory effects of SP in cultured neurons are difficult to reconcile with the lack of behavioral responses to local applications of SP. However, excitatory actions of SP may underlie responses of neurons innervating some internal organs, for example, bladder (74).

Opposing modulation of T-type channel activity by the latest generation of selective T-type channel modulators, ST101 (potentiator) and Z944 (inhibitor) confirmed the role of this channel in the control of sensory neuron excitability (Fig. 8 and Supplementary Figs. S7 and S8). Accordingly, a peripheral injection of ST101 induced moderate “spontaneous” nociception and increased sensitivity to both light and noxious touch, whereas Z944 reduced heat and touch sensitivity (Fig. 9); these effects are consistent with the analgesic efficacy of Z944 (31).

Importantly, we now show that both S9SP and Z944 significantly attenuate nociception induced by a peripheral injection of BK and *in vivo* knock-down of Ca_V_3.2 in DRG reduces such attenuation (Fig. 10). Co-application of Z944 with M-channel opener RTG produced a similar anti-nociceptive effect as that of S9SP (Fig. 10).

**FIG. 11.**
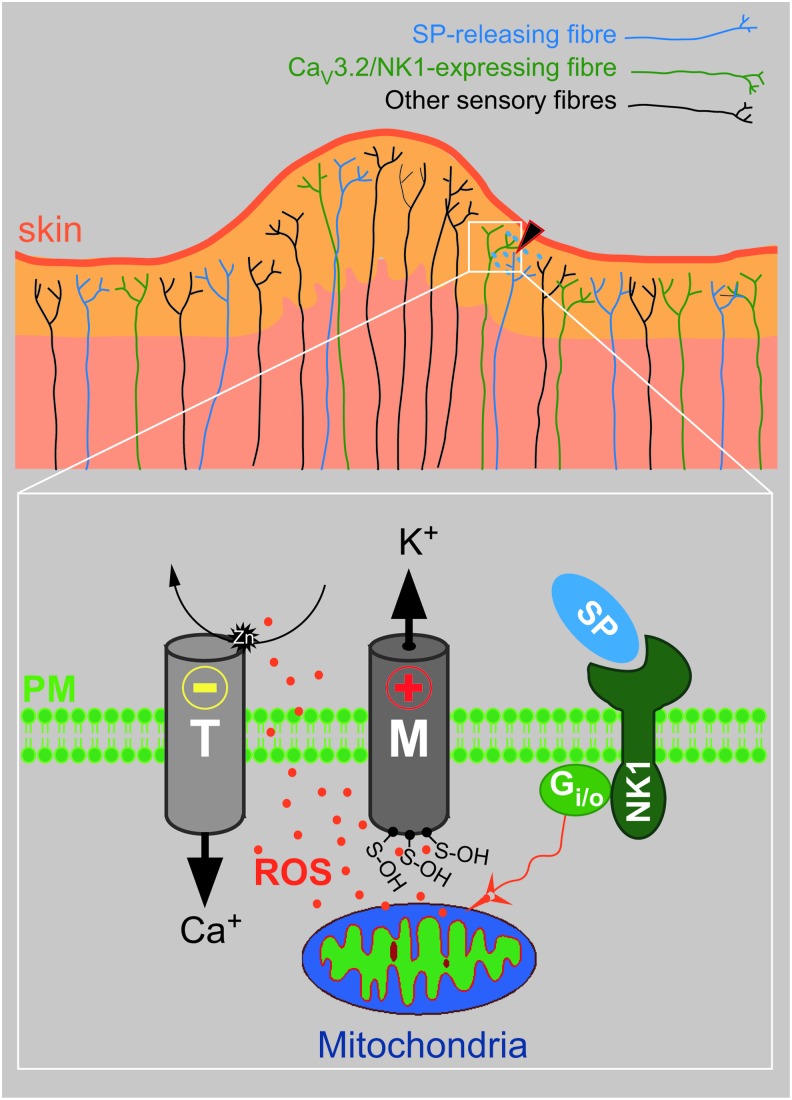
**Schematic of the proposed peripheral anti-nociceptive signaling** ***via*** **NK1 receptors.** Local tissue damage excites peptidergic nociceptors that release SP into the tissue damage area. Acting at the NK1 receptors expressed in the neighboring fiber endings (potentially including the SP-releasing fibers as well), SP triggers mitochondrial ROS release and redox-dependent inhibition of T-type Ca^2+^ channels and potentiation of M-type K^+^ channels, resulting in hyperpolarization and reduced excitability of the fibers. ROS, reactive oxygen species. To see this illustration in color, the reader is referred to the web version of this article at www.liebertonline.com/ars

Since both M-type (33) and T-type (present study) channels are modulated by SP (although in opposite directions) *via* a signaling cascade that shares common initial steps, we suggest that both M-channel augmentation and T-type channel inhibition contribute to the redox-mediated anti-nociceptive action of SP in peripheral nerves (Fig. 11). We hypothesize that since peptidergic nociceptive fibers release SP locally in response to noxious stimulation, this neuropeptide may exert local endogenous analgesia in an autocrine/paracrine fashion by simultaneous inhibition of T-type Ca^2+^ channels and augmentation of M channels in nociceptors.

Although we have previously shown that SP does induce mitochondrial ROS release in DRG neurons (33), the release of ROS from other, non-neuronal NK1-expressing cells surrounding the site of tissue damage cannot be excluded (particularly in the *in vivo* experiments), especially given the fact that the redox-sensitive site in T-type channels (but not M-type channels) is located extracellularly. Additionally, activation of NK receptors in DRG may result in the release of other substances that can modulate T-type channel activity, such as thioredoxin (4) or H_2_S (14, 15). Interestingly, both factors inhibit Ca_V_3.2 channels by interfering with their extracellular zinc-binding site (4, 14, 15).

Finally, although we could not detect any changes in intracellular or extracellular zinc levels in response to SP *in vitro*, one cannot exclude that some degree of redox-mediated zinc liberation from intracellular ligands, such as metallothioneins (28), or even extracellular zinc-bound proteins could occur (especially in the *in vivo* situation). Such zinc release could potentially contribute to the SP-mediated T-type channel inhibition, although in the case of intracellular zinc, it would need to be transported to the extracellular space to be able to affect T-type channel activity.

Clearly, further research is needed to address these intriguing questions; nevertheless, the present study firmly establishes redox-dependent modulation of T-type Ca^2+^ channels in nociceptive neurons as a mechanism contributing to the acute anti-nociceptive action of SP in the peripheral nociceptive system.

## Materials and Methods

### Cell culture, transfections, complementary DNA constructs, and chemicals

DRG neurons from adult male Sprague–Dawley rats (170–180 g) were dissociated as previously described (13, 29) and cultured in DMEM supplemented with GlutaMax I, 10% fetal calf serum, penicillin (50 U/ml), and streptomycin (50 μg/ml) on glass coverslips that were coated with poly-d-lysine for 2–5 days in a humidified incubator (37°C, 5% CO_2_). No growth factors were added to the culture media. HEK 293 cells were cultured in the same DMEM media; cells were passaged every 2 days. Human NK1 receptor (GenBank accession No. AY462098) complementary DNA (cDNA) was from the Missouri Science and Technology cDNA Resource Center. WtCa_V_3.1 (GenBank accession No. AF027984), wtCa_V_3.2 (GenBank accession No. AF051946), Ca_V_3.1Q172H, and Ca_V_3.2H191Q were kindly provided by Dr E. Perez-Reyes (University of Virginia). HEK293 cells were transfected using Lipofectamine 2000 (Invitrogen) according to the manufacturer's instructions. ST101 was synthesized in house; Z944 was from Toronto Research Chemicals; PTX was from Enzo Life Science; and FluoZin-3AM was from Life Technologies. All other chemicals were from Sigma.

### siRNA gene silencing

To knock down Ca_V_3.2, DRGs were dissociated as described (13); immediately after dissociation, DRG pellets were resuspended in 900 μl DMEM (without GlutaMax I, serum or antibiotics), to which a mixture of 9 μl Lipofectamine RNAiMAX (Invitrogen) and either a mix of three anti-Cacna1h Stealth siRNAs (RSS350285, RSS350286, RSS350287; Life Technologies; 200 n*M* each) or a scrambled control oligo (Stealth RNAi^™^ siRNA Negative Control; Life Technologies; 200 n*M*) were added immediately before resuspension. Cultures were than plated in 50 μl droplets onto glass coverslips that were coated with poly-d-lysine and incubated for 5–6 h; the transfection media were then replaced with full DRG culture medium. Patch-clamp recordings were conducted 24–48 h after the transfection. The following primers were used for reverse transcription polymerase chain reaction (RT-PCR) analysis:

Cacna1h sense: 5′-TGCCCACGGAGTCTATGAGT-3′
Cacna1h AS: 5′-GTTGTAGGGGTTCCGGATGT-3′
Gapdh sense: 5′-GACATGCCGCCTGGAGAAAC-3′
Gapdh AS: 5′-AGCCCAGGATGCCCTTTAGT-3′

To knock down G_i/o_ or G_q/11_ Gα subunits, Silencer Select RNAi siRNA (Invitrogen) oligonucleotides were used; the DRG dissociation and transfection were performed as described earlier. A mixture of the siRNA against G_q_ and G_11_ or against G_i1-3_ and G_o_ was co-transfected. The following siRNA oligos were used:

Anti-Gnaq sense: 5′-UGACUCUACCAAAUACUAUtt-3′
Anti-Gnaq AS: 5′-AUAGUAUUUGGUAGAGUCAga-3′
Anti-Gna11 sense: 5′-GCAUCGCUACAGUAGGCUAtt-3′
Anti-Gna11 AS: 5′-UAGCCUACUGUAGCGAUGCgg-3′
Anti-Gnai1 sense: 5′-GAAGCUGUUCGAUAGCAUAtt-3′
Anti-Gnai1 AS: 5′-UAUGCUAUCGAACAGCUUCat-3′
Anti-Gnai2 sense: 5′-CAGAGUGACUAUAUCCCUAtt-3′
Anti-Gnai2 AS: 5′-UAGGGAUAUAGUCACUCUGtg-3′
Anti-Gnai3 sense: 5′-CAAUGAUUCUGCUUCAUAUtt-3′
Anti-Gnai3 AS: 5′-AUAUGAAGCAGAAUCAUUGag-3′
Anti-Gnao sense1: 5′-GCAGAUGAAGAUCAUCCAUtt-3′
Anti-Gnao AS1: 5′-AUGGAUGAUCUUCAUCUGCtt-3′
Anti-Gnao sense2: 5′-CCUCCACUUCAGGCUGUUUtt-3′
Anti-Gnao AS2: 5′-AAACAGCCUGAAGUGGAGGtt-3′
The following primers were used for RT-PCR analysis:
Gnaq sense: 5′-TCCCAGAATATGATGGACCCC-3′;
Gnaq AS: 5′-CGGATGTTCTCCGTGTCTGT-3′.
Gna11 sense: 5′-ACAAGGCCAATGCACTCCTG-3′;
Gna11 AS: 5′-GGTCCACGTCCGTCAAGTAG-3′.
Gnai1 sense: 5′-GGATGATGCTCGCCAACTCT-3′;
Gnai1 AS: 5′-CATTCAGGTAGTACGCCGCC-3′.
Gnai2 sense: 5′-TACACAGGGGCCAACAAGTAT-3′;
Gnai2 AS: 5′-CTCACAGGTAGTGGGGAGCA-3′.
Gnai3 sense: 5′-TGTAGGTGGCCAAAGATCCG-3′;
Gnai3 AS: 5′-TGCATTCGGTTCATTTCCTCG-3′.
Gnao1 sense: 5′-AAAGCAAAAACCGCTCACCC-3′;
Gnao1 AS: 5′-CATGAAGCAGTCAAATAGGTTGC-3′.

### Electrophysiology

All recordings were made using Multiclamp 700B amplifier in combination with pCLAMP 10.4 software (Molecular Devices). Voltage-clamp recordings from DRG neurons were made using whole-cell patch clamp. The standard bath solution contained the following (in m*M*): 150 TEA-Cl; 2.5 CsCl; 2.5 CaCl_2_; 10 HEPES; 0.5 MgCl_2_; and 10 glucose (pH 7.4 adjusted with CsOH). The standard pipette solution contained the following (in m*M*): 110 CsCl; 3 MgCl_2_; 10 EGTA; 10 HEPES; 3 Mg-ATP; and 0.6 GTP (pH 7.4 adjusted with CsOH). The access resistance was typically within 4–8 MΩ. T-type calcium currents (LVA) were measured by 50 ms square voltage pulses to −40 mV from a holding potential of −90 mV. HVA currents were measured by 200 ms square voltage pulses to 0 mV from a holding potential of −90 mV.

In recordings from HEK 293 cells, amphotericin B-perforated patch-clamp technique was used (13). The pipette solution contained the following (in m*M*): 155 CsCl; 10 HEPES; 1 EGTA; and 4 MgCl_2_ supplemented with amphotericin B (250 μg/ml), pH 7.4 adjusted with CsOH. The bath solution was the same as for DRG neuron recordings. The access resistance was typically within 8–12 MΩ.

Voltage-clamp recordings were sampled at 4 kHz. Series resistance was compensated online by 50%; the voltage error due to series resistance in our recordings did not exceed 6 mV and typically was within ∼2 mV.

In experiments with H_2_O_2_, 3% agar-salt (3 *M* KCl) bridges were used. Concentration dependence of Ca_V_3.2 channel inhibition by zinc was fit to the two-component logistic function:

\begin{align*}
 { \frac { { I_ { max } } - I }  { { I_ { max } } } } = { \rm { } } { \frac { { \frac { { N_ { \left. { 0 ( Max1 } \right) } } }  { { N_0 } } } }  { 1 + { { \left( { { \frac { I { C_ { \left. { 50 ( 1 } \right) } } }  { \left[ { Z { n^ { 2 + } } } \right] } } } \right) } ^ { nH } } } } + { \rm { } } { \frac { { \frac { { N_ { \left. { 0 ( Max2 } \right) } } }  { { N_0 } } } }  { 1 + { { \left( { { \frac { I { C_ { \left. { 50 ( 2 } \right) } } }  { \left[ { Z { n^ { 2 + } } } \right] } } } \right) } ^ { nH } } } } ,
\end{align*}

where *N*_0_ is the total amount of zinc-binding sites, *N*_0(max1)_ is the amount of high-affinity binding sites, *N*_0(max2)_ is the amount of low-affinity binding sites, *I*_max_ is the maximal Ca_V_3.2 peak current amplitude, IC_50_ is the [Zn^2+^] necessary for half-maximal inhibition, and *nH* is the Hill coefficient. The concentration dependency of the S9SP effect was fit to the one-component logistic function:

\begin{align*}
{ \rm{y}} = {{ \rm{A}}_{ \rm{2}}} + ( {{ \rm{A}}_{ \rm{1}}} - {{ \rm{A}}_{ \rm{2}}} ) / ( {{ \rm{1}} + {{ ( {x / {x_0}} )}^{nH}}} ),
\end{align*}

where y is the current inhibition; A_1_ and A_2_ are the maximum and minimum effects, respectively; *x* is the S9SP concentration; and *nH* is the Hill coefficient.

Neuronal excitability was studied using the amphotericin B-perforated patch-clamp current technique (13). The extracellular solution contained the following (in m*M*): 160 NaCl, 2.5 KCl, 5 CaCl_2_, 1 MgCl_2_, 10 HEPES, and 8 Glucose. The intracellular solution contained the following (in m*M*): 145 KCl, 15 NaCl, 5 MgCl_2_, 10 HEPES, and 250 μg/ml Amphotericin B. The access resistance was typically within 8–12 mΩ. APs were generated by an injection of 200 pA current for 1 s from a holding current of 0 pA. Rheobase was determined from a train of current steps from 0 to 400 pA delivered in 20-pA increments. Current clamp recordings were sampled at 12.5 kHz. Bridge balance adjustments were not applied, since only relative changes in E_rest_, AP number, and rheobase were investigated.

### Measurement of zinc concentrations

The concentrations of zinc in experimental solutions were determined using an automated Ion Channel Reader 8000 flame atomic absorption spectrometer (Aurora Biomed) according to the manufacturer's instructions. To test whether SP induces zinc release from the cultured DRG cells, DRG cultures were grown on 10 mm round coverglass in 24-well plates; cultures were washed with extracellular solution (EC) and incubated in 0.5 ml EC that was supplemented with 1 μ*M* S9SP for 2–10 min; and supernatants were collected and analyzed for zinc content.

### Zinc imaging

Changes in the intracellular zinc levels were evaluated using fluorescence imaging. DRG cultures were loaded with FluoZin-3AM (5 μ*M* for 30 min at 37°C in the presence of 0.02% pluronic F-127). Cells were washed with extracellular bath solution composed of the following (in m*M*): 160 NaCl; 2.5 KCl; 2 CaCl_2_; 1 MgCl_2_; and 10 HEPES (pH 7.4 adjusted with NaOH); they were imaged using a Leica SP5 fluorescence imaging system assembled on a Leica DMI6000 microscope and illuminated with 488 nm light for 1200 ms (400 Hz) with a 2 s interval. Solutions were applied using a gravity perfusion system.

### Behavioral assays

Sprague–Dawley rats (body weight, 170–180 g) were grouped randomly and allowed to acclimate for at least 30 min in a transparent observation chamber before the experiment. Animals received a 50-μl intraplantar injection of ST101 (0.001–10 nmol/site), BK (10 nmol/site), RTG (1–10 nmol/site), Z944 (0.05–5 nmol/site), SP (0.5–5 nmol/site), or saline, respectively, into the right hind paw. For evaluation of the acute “spontaneous” pain, animals were video-recorded for 30 min after the injection and protective (“nocifensive”) behavior was analyzed as time spent licking, biting, lifting, and flinching the injected paw. The analyses were performed by an observer who was unaware of treatment allocations. In the pre-injection experiments, the right hind paw of the animal received a pre-injection of 50 μl Z944 (0.05–0.5 nmol/site), RTG (1–10 nmol/site), SP (0.5 nmol/site), Z944 (0.05–0.5 nmol/site)+RTG (1 nmol/site), or saline; 5 min later, on the same paw, the animal received a further 50-μl injection of BK (10 nmol/site) together with the pre-injection drug mix. Nocifensive behavior was analyzed as described earlier.

For measurement of thermal hyperalgesia, the change in the latency of hind-paw withdrawal in response to noxious heat was recorded using the Hargreaves’ plantar method (Ugo Basile) at 8 min after the hind-paw injection of drugs; the heat source was set to 25% of the maximal intensity.

For measurement of sensitivity to gentle touch and mechanical hyperalgesia, two methods were used. (i) Cotton swab test was performed as previously described (53). Briefly, rats were allowed to acclimate in a cage with a wire mesh bottom for at least 30 min and then were manually probed with a “cotton swab” pulled from a Q-tip (to approximately three times the original size). A swab was applied in a sweeping motion underneath the rats’ paws, and the withdrawal responses were recorded. Five sweeps were applied in each test, with at least 10 s between each sweep; withdrawal score was calculated as a percentage of positive responses. (ii) The von Frey test was performed using a calibrated range of filaments with different bending forces (2, 4, 6, 8, 10, and 15 g; Stoelting Co.). A single filament was applied perpendicularly to the plantar surface of the hind paw for five times, with an interval of 5 s, and it was scored for withdrawal responses. Each force was probed five times, the number of withdrawals out of five trials was counted, and the percentage of withdrawal responses was calculated. Both types of assays were performed 8 min after the hind-paw drug injection.

### Intrathecal administration of ODNs targeting Ca_V_3.2 subunit

The *in vivo* Ca_V_3.2 knock-down was performed as described earlier (3) by using the same ODN sequences that were synthesized by SBS Genetech. AS ODNs had the following sequence: CCACCTTCTTACGCCAGCGG. The mismatch ODNs of the following sequence were used as a negative control: TACTGTACTTGCGAGGCCAC. The mismatch ODN was not complementary to any known nucleotide sequence. ODNs were reconstituted in saline before injection. ODNs (12.5 μg/rat in 10 μl of saline) or saline were injected intrathecally (i.t.) between the L5 and L6 dorsal spinous processes as described earlier (3, 42) under isoflurane anesthesia. The treatment was repeated twice daily for 4 days. The pain tests were performed on the next day after the last injection. After behavioral testing, animals were humanly sacrificed; lumbar DRGs were extracted and analyzed for the knock-down efficiency by comparing the *Cacna1h* mRNA levels in lumbar (L4 to L6) DRGs of the saline-injected, mismatch ODN-injected, and AS ODN-injected animals using RT-PCR. The same *Cacna1h* and *Gapdh* primers as in siRNA experiments were used for RT-PCR.

### Reagents

Signaling peptide bradykinin (Arg-Pro-Pro-Gly-Phe-Ser-Pro-Phe-Arg; BK; Sigma); NK1-selective tachykinin receptor agonists [Sar9]-Substance P (Arg-Pro-Lys-Pro-Gln-Gln-Phe-Phe-Sar-Leu-Met-NH_2_; S9SP; Sigma) and [Sar^9^, Met(O_2_)^11^]-Substance P (Arg-Pro-Lys-Pro-Gln-Gln-Phe-Phe-Sar-Leu-Met[O_2_]-NH2; S9M11SP; synthesized by SBS Genetech); NK1-selective antagonist CP-99994 (Sigma); reducing agent DTT (Sigma); G_i/o_ pathway inhibitor PTX (Sigma); M-type channel opener RTG (synthesized in house); VGCC blocker MIB (Sigma); T-type specific Ca^2+^ channel blocker Z944 (synthesized in house); cognitive enhancer and T-type Ca^2+^ channel activator ST101 (synthesized in house); zinc chelator TPEN (Sigma); and protein kinase C inhibitor, BIM I were used as reagents.

### Statistics

All data are given as mean ± standard error of the mean. Differences between groups were assessed by Student's *t* test (paired or unpaired, as appropriate) or one-way analysis of variance (ANOVA) with Bonferroni correction. Behavioral experiments were analyzed using Kruskal–Wallis ANOVA; difference between groups was analyzed with Mann–Whitney test. The effects of the drugs on neuronal excitability were analyzed using either paired *t* test or paired Wilcoxon signed ranks test (for data sets that failed normality test). The differences were considered significant at *p* ≤ 0.05. Statistical analyses were performed using Origin 8.6 (OriginLab Corporation).

## Supplementary Material

Supplemental data

Supplemental data

Supplemental data

Supplemental data

Supplemental data

Supplemental data

Supplemental data

Supplemental data

Supplemental data

## Abbreviations Used

ANOVA: analysis of variance
AP: action potential
AS: antisense
BIM I: bisindolylmaleimide I
BK: bradykinin
CNS: central nervous system
CP-99994: (2S,3S)-3-(2-Methoxybenzylamino)-2-phenylpiperidine dihydrochloride
DMEM: Dulbecco's modified Eagle medium
DRG: dorsal root ganglion
DTT: dithiothreitol
E_rest_: resting membrane potential
ETC: electron transport chain
H_2_S: hydrogen sulfide
HEK: human embryonic kidney
HVA: high-voltage activated
LVA: low-voltage activated
MIB: mibefradil
mRNA: messenger RNA
NK: neurokinin
ODN: oligodeoxynucleotide
PTX: pertussis toxin
ROS: reactive oxygen species
RTG: retigabine
RT-PCR: reverse transcription polymerase chain reaction
S9M11SP: [Sar^9^, Met(O_2_)^11^]-Substance P
S9SP: [Sar^9^]-Substance P
SP: substance P
ST101: spiro[imidazo[1,2-a]pyridine-3,2-indan]-2(3H)-one
TPEN: N,N,N′,N′*-*Tetrakis(2-pyridylmethyl)ethylenediamine
TRPV1: transient receptor potential cation channel subfamily V member 1
VGCC: voltage-gated Ca^2+^ channel
Z944: N-[[1-[2-(tert-butylamino)-2-oxoethyl]piperidin-4-yl]methyl]-3-chloro-5-fluorobenzamide
ZnPy: zinc pyrithione
